# Self-motion illusions from distorted optic flow in multifocal glasses

**DOI:** 10.1016/j.isci.2021.103567

**Published:** 2021-12-08

**Authors:** Yannick Sauer, Malte Scherff, Markus Lappe, Katharina Rifai, Niklas Stein, Siegfried Wahl

**Affiliations:** 1 Institute for Ophthalmic Research, University of Tuebingen, Tuebingen 72076, Germany; 2 Department of Psychology & Otto Creutzfeldt Center for Cognitive and Behavioral Neuroscience, University of Muenster, Muenster 48149, Germany; 3 Carl Zeiss Vision International GmbH, Aalen 73430, Germany

**Keywords:** Neuroscience, Systems neuroscience, Sensory neuroscience

## Abstract

Progressive addition lenses (PALs) are ophthalmic lenses to correct presbyopia by providing improvements of near and far vision in different areas of the lens, but distorting the periphery of the wearer's field of view. Distortion-related difficulties reported by PAL wearers include unnatural self-motion perception. Visual self-motion perception is guided by optic flow, the pattern of retinal motion produced by self-motion. We tested the influence of PAL distortions on optic flow-based heading estimation using a model of heading perception and a virtual reality-based psychophysical experiment. The model predicted changes of heading estimation along a vertical axis, depending on visual field size and gaze direction. Consistent with this prediction, participants experienced upwards deviations of self-motion when gaze through the periphery of the lens was simulated, but not for gaze through the center. We conclude that PALs may lead to illusions of self-motion which could be remedied by a careful gaze strategy.

## Highlights

• Multifocal lenses impair vision of spectacle wearers with gaze-dependent distortions
• A model of heading perception from distorted optic flow suggest a misperception
• Heading perception was tested with a virtual reality-based simulation of distortions
• Distortions lead to gaze direction-dependent illusions in perceived vertical heading

## Graphical abstract

**Figure:**
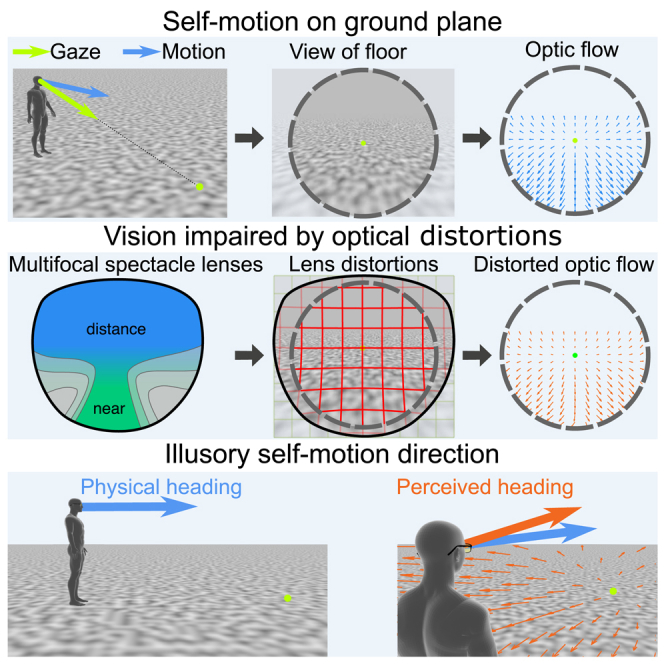

## Introduction

**Figure 1:**
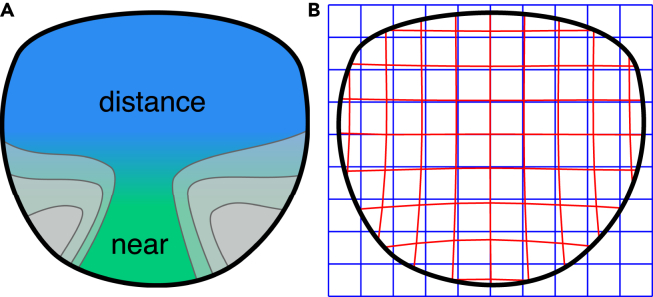
Schematic illustration of a PAL (A) The lens has a zone optimized for distance vision in the upper and a zone optimized for near vision in the lower part. The gradient of color represents a progressive change of optical power between the two zones. As a side effect, the power gradient leads to astigmatic blur in the left and right periphery, indicated by the gray areas. (B) PALs distort the visual field of the wearer. The blue mesh represents object points in the visual field. PAL distortions displace the perceived position as illustrated by the red mesh. Prominent distortions are in the lower periphery.

With increasing age, accommodation capabilities of the human eye decrease and it becomes harder to focus on nearby objects (Atchison, 1995). Progressive addition lenses (PALs) are a common treatment method using a special kind of spectacle lens design with complex optical properties. PALs have two regions of different optical power for far and near vision (Meister and Fisher, 2008a) (Figure 1A). The far zone in the upper area of the lens corrects far vision, whereas the near zone in the lower area of the lens has additional optical power to reduce accommodative demand when focusing on close objects. Between these two zones, there is a progressive increase in optical power which inevitably produces unwanted astigmatism (Sheedy et al., 2005). This causes blur and skew distortions in the periphery. Thus, the wearers of these lenses have to cope with spatially varying distortions of the visual field. Figure 1B shows a simulation of a progressive addition lens' optical distortions.

When wearers of PALs move their eyes, these distortions are not fixed in the visual field but change with gaze direction. During central view through the upper area of the lens that is designed for far vision, the distortions are visible mainly in the visual field's periphery. Alternatively, gaze direction through the lower left or right area of the lens leads to substantial distortions in the visual field center.

Optical distortions can have negative influences on visual perception, including form, motion, and distance estimations. Many PAL wearers report a perception of unnatural motion of the environment (swim effect) (Meister and Fisher, 2008b), even causing nausea for some of them (Alvarez et al., 2009). A considerable number have problems with tripping while wearing PALs (Johnson et al., 2007; Lord et al., 2002). Because some wearers cannot adapt to the distorted perception, they reject PALs entirely. We wondered whether the difficulties might be related to distortions of the visual motion experienced during locomotion.

Walking through a rigid environment forms moving patterns of light on the retina of the observer. These patterns are called optic flow and they contain information about amplitude and direction of motion relative to the surroundings and about the appearance of the scene (Gibson, 1950). Typically optic flow is described as a two-dimensional vector field, so-called optic flow field, which carries information about the retinal placement and direction of each flow element. The human visual system is able to extract information from optic flow for the control of self-movement (Warren and Hannon, 1988; Lappe et al., 1999; Warren et al., 2001), the perception of the environmental structure (Andersen, 1989), the prevention of collision (Sidaway et al., 1996), and the estimation of traveled distance (Lappe et al., 2007).

For a pure translational motion of the observer, e.g., walking with gaze fixed on the horizon, optic flow consists of a radial pattern expanding from a single point in the direction of the translation, the focus of expansion (FoE). However, such a static gaze is a rare occurrence during self-motion. During walking, humans look around, often at the ground in front, in order to check for obstacles and find good locations for foot placement (Hollands et al., 1995; Patla and Vickers, 1997; Calow and Lappe, 2008; ‘t Hart and Einhauser, 2012; Matthis et al., 2018), or to adjust gaze toward a new destination when changes on the planned path occur (Hollands et al., 2002). In such cases, rotational eye movements stabilize the gaze on objects in the environment via smooth pursuit (Niemann et al., 1999) or eye rotation reflexes (Miles and Busettini, 1992; Lappe et al., 1998; Angelaki and Hess, 2005; Matthis et al., 2018). Because of the rotation of the eye, the resulting flow structure on the retina is no longer a radial pattern but resembles a spiral with the center near the fovea (Warren and Hannon, 1990; Lappe and Rauschecker, 1995; Lappe et al., 1999). Such patterns, despite lacking retinal motion at the visual field's center, contain a large variety of motion directions at low speed in the parafoveal area. The peripheral visual field, in contrast, features much higher speeds in more radially distributed directions (Calow and Lappe, 2007). The visual system is able to recover from such retinal flow (Warren and Hannon, 1990; Lappe et al., 1999; Bremmer et al., 2010). PAL glasses, however, would produce additional distortions in this pattern, depending on how gaze is directed through the lens.

**Figure 2:**
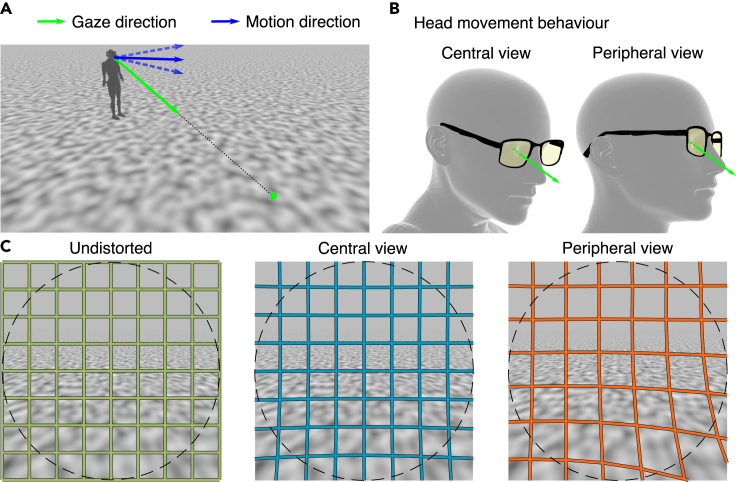
Experimental setup (A) Visualization of the self-motion scenario used in this study: Gaze (green) is downwards onto the floor and off to the side. Self-motion (blue) consists of a translation over the ground. In experimental conditions, translation could also be slightly downward or slightly upward with respect to the ground (dashed blue lines). (B) Two different head orientations while fixating a point on the ground. In the central view condition the observer's line of sight is through the center of the lens and the head is tilted downward and to the side. In the peripheral view condition, the head remains at level and gaze is through the periphery of the lens. (C) The visible scene of the moving environment is the same in all conditions, but distortions, illustrated by colored meshes on top, differ between central view and peripheral view. The dashed circle indicates a circular field of view with radius $50^{\circ }$ as presented in the VR experiments.

When a person aims to fixate a point on the floor during walking, various combinations of head and eye movements are suitable to execute this task. One possible way to do so is to keep the head orientation straight in walking direction and direct the eye downward to fixate the ground (Figure 2B, right). Alternatively, one might tilt the head downward while keeping the orientation of the eye straight with respect to the head (Figure 2B, left). Although these two behaviors result in the same structure of optic flow in normal eyes, they lead to different distortions for PAL wearers: When the eye is directed downwards but the head remains level, gaze is through the peripheral, more distorted part of the lens. Therefore, distortion occurs in the center of the visual field. In contrast, when the head, and therefore the PAL glasses, are moved, gaze is directed through the less distorted central area of the PAL and the distortion occurs mainly in the visual periphery.

Distortions alter the optic flow a PAL wearer receives on the retina, possibly resulting in an internal misperception regarding the own movement. Our study aimed to investigate the influence of PAL distortions on heading perception and to determine differences in perception between the central and peripheral views in a typical self-motion scenario. We had two goals. First, we wanted to determine how PAL distortions would be expected to affect heading estimates. For this, we first created PAL distorted flow fields and then used computer simulations of an established model of heading perception in humans to estimate the expected effects on heading. Second, we wanted to determine if PAL distortions lead to misperceptions of self-motion in human observers. For this, we tested heading estimation in human participants in a virtual reality (VR) simulation of PAL distortions along the axis that provided the strongest predicted misperception in the model simulations.

## Results

### Simulations of PAL distortions in retinal flow fields

We simulated a translation across a ground plane while fixating a point on the floor next to the path (Figure 2A), a scenario that occurs naturally during walking (Hollands et al., 1995; Patla and Vickers, 1997; Calow and Lappe, 2008; ‘t Hart and Einhauser, 2012; Matthis et al., 2018). We considered two distortion conditions corresponding to central view and peripheral view through the PAL (Figure 2C). Optic flow fields were calculated for the motion scenario with and without distortions using a ray tracing approach in which for each point in the scene the distorted and undistorted positions in the image plane are calculated followed by calculation of the (distorted) optical motion in the image plane (see STAR Methods).

**Figure 3:**
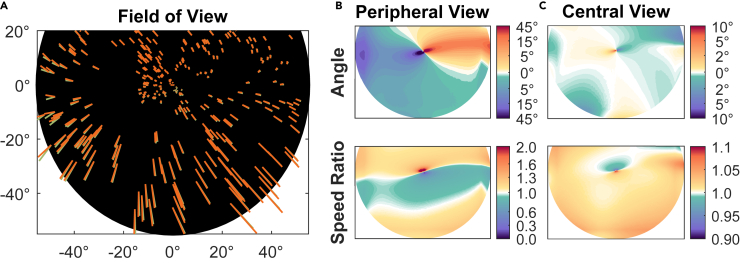
Changes in flow fields because of distortions (A) Example flow fields used in the simulations for a translation parallel to the ground to the left of the fixation point. Green arrows show the undistorted flow, orange arrows show the flow distorted because of the peripheral view condition. (B) The top panel shows the angle between flow vectors at all points in the visual field for the peripheral condition. A position colored in the yellow-red spectrum indicates an anti-clockwise turn from an undistorted vector to the distorted vector there. The bottom panel shows changes in speed as the ratio of the speed of the distorted vector to the speed of the undistorted vector. Values greater than 1 indicate an increase in speed at that position. (C) Directional and speed changes because of the central gaze condition. The color scales are limited because of the undistorted flow in the central area almost vanishing, limiting its validity as a reference there.

Panel A in Figure 3 shows an example of a superposition of the peripheral view distorted (orange arrows) on top of the undistorted (green arrows) retinal flow field. In this particular simulation, the direction of self-motion (heading) was to the left. The overall pattern appears very similar in both cases and exhibits the typical spiral flow field structure for the fixation of a ground element (Warren and Hannon, 1990; Lappe and Rauschecker, 1995; Lappe et al., 1999). Differences between the distorted (orange) and undistorted (green) flow can be seen in the lower left area but appear to be rather small. However, panel B shows how the distortion changes direction (angle) and speed (ratio) across the visual field and reveals that there are large areas of systematic differences between the distorted and the undistorted flow. The parafoveal area exhibits directional changes of the flow, clockwise on one side and anti-clockwise on the other side of the fovea, as well as increases and decreases of speed in the upper and lower parafoveal fields. Owing to the typically low flow speeds in this area, even a small distortion may already cause a different flow perception, impacting the spiral structure emerging from the center. Panel C shows the differences in direction and speed in the central view condition. They exhibit a different retinal distribution than in the peripheral view condition and are overall smaller. The quantitative analysis of both distortions shows that they do not act as random noise to the flow field but impose a systematic and continuous pattern of changes to the optic flow field. These systematic differences in flow, though small in value, might lead to misestimations or increased variability in heading estimates. Owing to the complexity of the flow field, however, it is not immediately clear which or how much distortions of self-motion perception may be expected. We therefore used computational modeling to better understand the potential implications of the optic flow distortion.

**Figure 4:**
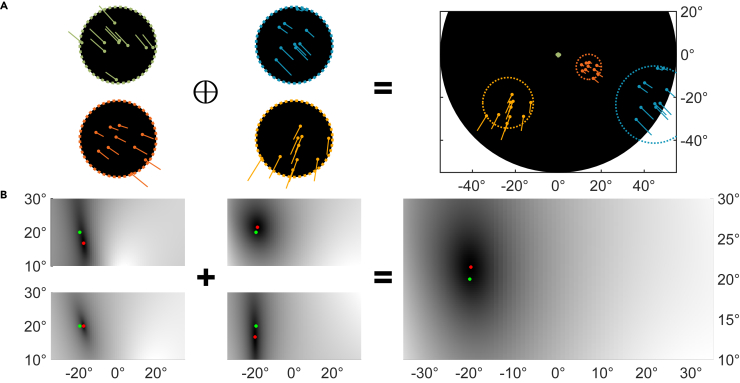
Visual description of the simulation process The simulation calculates the map of heading likelihoods for a particular flow field with a defined heading and gaze direction: (A) Circular patches (four are shown in this example) were placed randomly inside the visual field with area size proportional to eccentricity. A total of 10 points per patch were selected randomly and their motion vectors were calculated according to the heading and gaze direction. Combining the patches yields the optic flow field for this heading direction (−20^∘^,20^∘^) in the peripheral distortion condition. (B) The likelihoods of all the heading directions, shown in retinal coordinates that are considered as an explanation for the small flow fields are presented as a gray-scale map. The darker a direction is displayed the more likely it is to explain the flow field. The combination of the ratings for the small and locally restricted flow fields yields a global estimate for heading direction. Red dots mark the most likely heading, i.e., the heading, which according to the model, explains the flow field best. The green dots indicate the true heading direction.

To estimate whether these distortions might influence heading estimation from retinal flow, we presented distorted flow fields to the method for heading recovery used in an established model of heading perception in humans (Lappe and Rauschecker, 1993; Lappe et al., 1999; Li et al., 2018). This model implements the subspace algorithm for heading estimation (Heeger and Jepson, 1992) that computes a likelihood map of heading space, i.e., a 2D map of potential heading directions in retinal coordinates representing the likelihoods with which each one of them explains a given flow field. Distortions of the flow field might influence the likelihood distribution in this map. Figure 4 shows an example of the likelihood distribution for a distorted flow field using a gray scale to indicate the likelihood of each heading direction. The model selects patches of flow (four of them are shown in the left part of panel A) from the flow field, similar to receptive fields of neurons in the visual motion pathway of the brain (Lappe et al., 1996). It then computes likelihood maps of the heading for each patch. The four small plots on the left of panel B show the likelihoods for the four patches in A. Dark areas indicate the most likely heading. Finally, the individual likelihood maps are averaged to compute a global likelihood map (right plot in panel B), in which the most-likely heading (red dot) is the model's heading estimation for the given flow field.

Because the most likely headings (dark area in Figure 4B) cover the true heading (green dots), the results of the simulation indicate that the heading perceived from this flow field is still close to the true heading. The vertical spread of the dark area indicates that PAL distortions might create uncertainty along a vertical axis because of the corresponding headings mainly differing in the vertical component. In case of an undistorted flow field the likelihood map would contain a single dark spot coinciding with the true heading and be colored white elsewhere.

We tested this prediction in a psychophysical experiment with flow fields including the different types of optical distortions resulting from the two distinct kinds of head orientation behavior, i.e., the central view condition and the peripheral view condition.

### Virtual reality experiment on distorted self-motion perception in humans

To study human perception under the influence of PAL distortions in an isolated experimental environment, we developed a simulation of optical distortions in VR (Stein et al., 2021). The PAL distortions of a Zeiss lens design (Carl Zeiss Vision GmbH, Aalen, Germany) were precomputed by ray tracing for different gaze directions. The virtual image was distorted to simulate PAL distortions for either the central view or peripheral view. For comparison, in a third condition the virtual image was undistorted. The self-motion scenario simulated movement across a virtual ground plane while fixating a point on the floor as in Figure 2. The self-motion was either parallel to the ground or contained a small upwards or downwards component between −3.5° and 3.5°. Participants reported their perceived direction of self-motion by indicating their perception of either *sinking in* or *lifting off* in each trial. The three distortion conditions were measured in one session in randomized trial order.

For each subject and each distortion condition, the vertical translation angle perceived as a motion parallel to the floor (point of subjective equality - PSE) was determined by fitting a psychometric function to the subjects' answers depending on the vertical translation angle. Then the difference of PSE between the distorted condition and the undistorted condition was calculated. This relative shift (ΔPSE) provided a measure for the influence of simulated distortions in the two view conditions.

**Figure 5:**
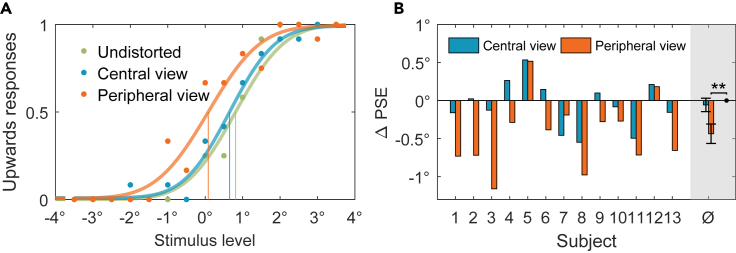
Experimental results (A) Percentage of upwards responses and psychometric fits for one example subject. The green data points were obtained in the undistorted trials. The blue and orange data points show the answers in the two distortion conditions for simulated central view and simulated peripheral view, respectively. All three datasets were fitted with a psychometric function. The 50% point of the fit function is taken as the point of subjective equality (PSE) (vertical lines). (B) Difference of the PSEs between the distortion conditions and the undistorted condition for all tested subjects. The rightmost bars are the mean value of Δ PSE with standard deviation as error bars. ∗∗p < 0.01.

Figure 5 A shows the answers and psychometric fits for all conditions for one example subject. There is a clear shift of the psychometric function in the case of simulated gaze through the periphery of the PAL compared to the undistorted condition. The PSE is 0.73^∘^ lower than in the undistorted condition. This subject perceived the translational movements more upwards than in the undistorted condition. In the central view condition, the PSE is similar to that of the undistorted condition. Figure 5B shows the change of PSE in the two distortion conditions relative to the undistorted condition across all subjects. In the peripheral view condition, there is a significant deviation (ΔPSE) from the undistorted condition (t test, p<0.01). On average, the angle perceived as a straight movement was 0.43^∘^ smaller than in the undistorted condition, i.e., the Δ PSE was negative. Thus, in the peripheral gaze condition participants reported a self-motion percept that appeared as if they were slightly lifting up from the ground when in fact the motion was parallel to the ground. For the simulated gaze through the center of the PAL, there is no significant difference between the distorted and undistorted cases.

### Model simulation of VR experiment

The results of the VR experiment showed that PAL distortions of the optic flow indeed produced misperception of self-motion along the vertical dimension. We next presented the same vertical heading task to the model in computer simulations to see whether the size of the misperception fitted the distortions of the flow fields. For these simulations, fifty flow fields with different random distributions of flow vectors were calculated for each heading direction. Then, these flow fields were distorted to create individual fields for the different distortion conditions. Next, the most likely heading direction according to the model was calculated using the subspace algorithm. Finally, to recreate the psychophysical procedure, model heading estimates were converted from a direction into upwards and downwards answers, depending on the sign of the vertical component relative to a direction parallel to the ground. In the case that the estimate was parallel to the ground, it was counted both for upwards and downwards answers. For each distortion condition, the percentage of answers upwards was computed for each vertical direction of the stimulus. Then, for each distortion condition, the vertical heading angle perceived as a motion parallel to the floor (PSE) was determined by fitting a psychometric function to the answers using the same fitting procedure as for the experimental analysis. Because the model in the undistorted condition always produced a PSE of 0 (i.e., it recovered the correct heading) the shift in PSE (ΔPSE) corresponds directly to the extracted PSE for the distortion conditions.

For the model simulations, the maximum field of view (FoV) was set to be circular with a radius of $55^{\circ }$. This matched the specifications of the VR headset we used for the experiment. However, the effective FoV when wearing an HMD is often smaller because parts of the screen are not visible because of individual adjustments in fitting the HMD on the head. Therefore, we also performed the simulation procedure with slightly reduced sizes of the FoV.

**Figure 6:**
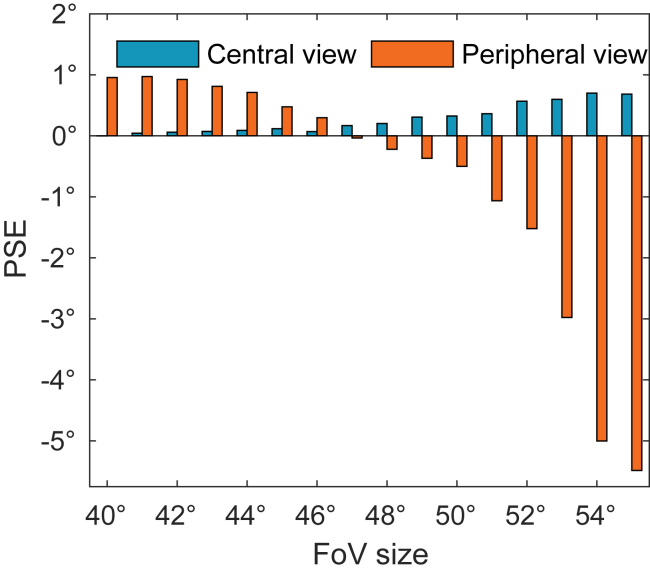
Simulation results Shift of PSE in the simulation results for the two distortion conditions for different simulated FoV sizes.

Figure 6 shows the PSE for the central and peripheral distortion conditions for effective FoV sizes between 40° and 55° radius.

For FoV sizes between 50° and 55°, the results show a negative PSE value for the peripheral view condition and a small positive value for the central view condition. This is consistent with the experimental findings which showed a significant negative PSE shift for the peripheral view condition and a much smaller PSE shift, not different from zero, for the central view condition.

Figure 6 furthermore shows that size and direction of the PSE depend on the size of the FoV. For FoVs smaller than 50^°^ the PSE in the central view condition further diminishes while the PSE in the peripheral view condition becomes positive, indicating a self-motion toward the ground. This dependence might be important in explaining individual data in the experiment and in considerations of the field of view in real PAL glasses.

## Discussion

### Summary of results

We investigated the influence of optical distortions of progressive addition lenses (PALs) on heading perception from optic flow for a translational movement across a ground plane while fixating a point on the floor, a typical walking scenario. We combined simulations of optic flow-based heading estimation and a VR-based psychophysical experiment. We compared two distortion conditions: first gaze through the center of the simulated progressive lens (central view condition) and second gaze through a point in the periphery of the lens (peripheral view condition). These two cases correspond to different combinations of gaze and head orientation. For the first condition, the observer would need to tilt the head downward while looking straight through the lens. For the second condition the head would remain level while the gaze would be directed downwards.

The resulting distortions of the progressive lens vary between the two scenarios because the periphery of the lens type we used induces considerably stronger distortions than the central area. In both experimental data and model simulations, we found that the central view condition produced small, if any, heading errors. The peripheral view condition, in contrast, produced heading estimates that appear as if one would slightly lift up from the ground.

### Discrepancies in experimental results

The experimental results had some variation between subjects in effect size as well as sign of Δ PSE, with two of them experiencing a positive shift in heading estimation in both distortion conditions. One might speculate that these variations could be explained by differences in effective FoV in the HMD, because model simulations also showed a dependence of the direction of heading estimate in the peripheral view condition on the size of the FoV. Although the rendered FoV of the HMD nominally spans 107^°^ horizontally and 108^°^ vertically (Musil, 2021), the effective FoV is usually smaller because parts of the screen are not visible. The FoV has a circular shape with a radius in the range of 45^°^to55^°^. The specific radius depends on individual factors, like physiological face properties and headset tightness, determining how close the eyes are to the HMD's lenses. These factors, together with a dependence of perceived heading on FoV might explain individual differences in our experiment.

On the other hand, some subject data do not match the effect of FoV in the model simulation. Although a positive shift of PSE in both distortion conditions fits to simulations with a small FoV, some subjects have a clear negative PSE in the central view condition - a result not predicted by the model. Experiments testing differently sized restricted FoVs are needed to better understand the influence of FoV and to possibly adapt the heading estimation model.

### Conclusion for optic flow processing

Participants in psychophysical experiments are able to estimate simulated heading directions from optic flow fields. How well this task is executed is coupled to the quality of the flow presented. Adding random noise to the flow increases the estimation errors (van den Berg, 1992; Foulkes et al., 2013). The PAL distortions, even though their influence on the global flow field pattern is subtle, add a form of noise to the flow fields and induce estimation errors as well. This estimation error is more systematic in the form of a heading bias along the vertical axis, an effect not reported from the addition of random noise to flow fields.

The PAL distortions led to continuous changes of speed and direction of flow field vectors that vary throughout the visual field. Regarding the typical spiral flow field structure, more radial in the periphery but rotational in the central area, the impact of the distortions may differ locally as well. A similar directional change in local flow in the periphery that is nearly unidirectional does not confound the spiral structure as much as such a directional change in local flow that curls around a single point in the center.

In our study, we found changes in heading estimations when the FoV changed in size, showing not only the influence of the extent of the flow field but also the aforementioned local differences in changes of flow structure. We conclude that for modeling heading estimation, it is necessary to consider location dependent flow processing and the presented model is a first step in establishing a simulation of heading estimation for such flow fields based on the local structures.

### Practical implications

Heading illusions as shown in this study would put visual perception in conflict with other self-motion perceptions and might relate to the ”swim effect” observed in novice wearers of PAL glasses (Meister and Fisher, 2008b) and contribute to discomfort and nausea shown by some PAL wearers (Alvarez et al., 2009). The amount of misperception differed depending on the area of the lens through which gaze was directed. For normal eye movement behavior with constantly changing gaze direction, this can produce motion perception characterized by a continuous change of perceived motion direction. In complicated walking scenarios, e.g., climbing stairs, misperception of heading changing during every eye movement can lead to complications with foot placement and tripping (Johnson et al., 2007), especially considering decreased capabilities of elderly people for heading control from optic flow (Berard et al., 2009).

An illusory, gaze-dependent perception of relative motion of the surrounding area is an often reported side effect for PAL wearers (swim effect) that still is only vaguely described (Han et al., 2003; Alvarez et al., 2009). Quantifying the very discomforting motion illusions by evaluating the distorted optic flow field would be a first step in objectively characterizing the swim effect, and even identifying differences between lenses. A PAL with large differences in the heading estimate for different gaze directions is expected to cause more discomfort. Therefore, optic flow-based analysis of PAL distortions might become a tool for lens design to improve the experience of PAL wearers by presenting distortions for more consistent optic flow perception.

Independent of design, our results suggest an advantage for heading estimation for the central view condition when distortions are mainly in the periphery. Wearers of progressive lenses should rather use the lens' central area while walking to perceive less distorted optic flow in the center of the visual field. This would lead to increased head movements for PAL wearers compared to other people, a change in behavior that was already found in a natural scenario (Rifai and Wahl, 2016).

Variable FoV size has to be considered not only in experimental conditions but also as a practical issue. The part of the visual field covered by a spectacle lens depends on frame size and distance to the eye. For a typical vertex distance of 12 mm, nodal point of the eye of 7.3 mm (Jones et al., 2016) and lens width between 40 mm and 60 mm, the horizontal FoV ranges between 92° and 115°. Points outside of the spectacle frame are not distorted. As a result there is a frame-dependent border between distorted and undistorted optic flow in the periphery, which might influence heading perception and might be an additional factor for discomfort because of inconsistencies in the optic flow pattern. Although our experiment did not investigate it, the FoV dependency of our simulation results suggest that frame size can also indirectly influence self-motion perception by determining the size of the distorted part of the visual field. To better understand the influence of FoV size, further experiments are needed which test perception for defined restricted areas in the visual field of changing size.

Most wearers of PAL spectacles, even if they initially experience motion misperceptions, adapt to their spectacles over a couple of weeks. Such adaptation plays an important role in the acceptance of progressive lenses. It has been shown that humans can adapt to distortions of the visual field. These include not only local simple geometric transformations (Jin et al., 2005) but also skew distortions as present for progressive lens wearers (Habtegiorgis et al., 2017) and spatially varying distortions (Sauer et al., 2020). Furthermore, skew distortion adaptation aftereffects show trans-saccadic transfer (Habtegiorgis et al., 2018), which is important in the context of distortion changes by eye movements. Aftereffects do not only appear as altered form features, but also in motion perception and even include interactions in which exposure to distorted form also influences motion perception (Rifai et al., 2020). Our experiments were designed to study motion misperceptions that occur during novel wearing of PAL glasses. It might be interesting to see how the adaptation process of PAL wearers can influence heading perception in future experiments.

### Outlook

In our simulation, we only considered translational motions as possible candidates for the distorted flow field. However, distortions might also induce misperceptions of rotational motion components. A perceived rotation could describe unnatural motion of the environment, reported by many PAL wearers. Therefore, an extension of our optic flow model to include rotations could help to further understand motion illusions in PAL wearers. Development of a quantification method of distortion-induced motion discomfort based on optic flow could help in the future design of progressive lenses.

### Limitations of the study

Because of the limited FoV of the VR headset, our experiment could not fully replicate the distortions visible to PAL wearers. The use of high-FoV headsets in the future could simulate a FoV similar to that covered by real spectacle frames. The measured effect might increase because of more visible distortions in the periphery. Regarding the everyday problems of PAL wearers, not only shifts of the most likely heading, but also other aspects of the optic flow field have to be considered. Distorted vision results in unnatural flow fields, which becomes clear from the heading likelihood maps: the distribution of likely headings is wide with no direction fitting perfectly to the distorted flow field. It is not clear to what extent the widened distribution of heading directions contributes to discomfort of PAL wearers. In addition, when self-motion does not suffice to explain the flow field entirely, illusory movement of the environment might be perceived to account for unexplained flow.

Quantification of the naturalness of distorted optic flow and measurements of the discomfort related to it are needed to cover this aspect. Furthermore, only one type of PAL was tested in this study. Different lenses with corrections for different refractive errors can differ in their distortions and therefore also in their influence on self-motion perception. Other distortions of the optic flow field might also induce horizontal heading illusions. Future studies might concentrate on the differences between different correction power or design parameters influencing the distortions.

## STAR★Methods

### Key resources table

**Table:** 

REAGENT or RESOURCE	SOURCE	IDENTIFIER
**Software and algorithms**		

MATLAB (R2020a)	MathWorks	RRID: SCR_001622
Unity (2019.4)	Unity Technologies	
Subspace algorithm	Heeger and Jepson (1992)	https://doi.org/10.1007/BF00128130
Model for heading estimation	This paper	https://github.com/MalteScherff/Self-motion-illusions-from-distorted-optic-flow-in-multifocal-glasses

### Resource availability

#### Lead contact

Further information and requests for resources should be directed to and will be fulfilled the lead contact, Yannick Sauer (yannick.sauer@uni-tuebingen.de).

#### Materials availability

This study did not generate new unique reagents.

### Method details

#### Ray tracing optical distortions

The lens design used for this study is a progressive addition lens (PAL) without optical correction in the far zone and with addition power 2dpt in the near zone. To simulate the distortions of this lens we calculated the projection of points on a virtual image plane in front of the lens onto the image plane behind the lens. The points on the virtual image plane with fixed distance *d* in front of the lens were defined on an equidistant grid. For each point (*x*,*y*) the perceived distorted position $\left(x_{d},y_{d}\right)$ through the lens is calculated by ray tracing. Figure 1 B shows an example of an undistorted image grid and a distorted image grid.

As a mathematical description of the distortion, we define a function F, which transforms a point (*x*,*y*) in the undistorted image plane to the point $\left(x_{d},y_{d}\right)$ in the distorted image, i.e. the point at which it would appear when looking through the lens.

(Equation 1) $$\left(\begin{array}{l} x_{d}\\ y_{d} \end{array}\right)=\left(\begin{array}{l} F_{x}\left(x,y\right)\\ F_{y}\left(x,y\right) \end{array}\right)$$

The two components of F and their derivatives were interpolated from a 21×21 ray traced grid using 2D linear interpolation in Matlab (The Mathworks Inc, Natick, MA, USA). Individual transformation functions were prepared based on individual distortion grids for the gaze directions tested in this study. In the central view condition, the simulated gaze during ray tracing was through the center of the PAL. For the peripheral view condition, the simulated gaze crossed the lens at 20^∘^ vertically downwards and ±20^∘^ horizontally.

#### Simulation of heading estimation

Our model simulations follow the subspace algorithm (Heeger and Jepson, 1992) as implemented in the population heading map model (Lappe and Rauschecker, 1995; Lappe et al., 1996). Here we describe how this model is applied to distorted flow. We begin by describing how the distorted flow field is calculated for a given heading and gaze direction and then describe the process and simulation for recovering heading from such a distorted flow field.

##### Calculation of the distorted optic flow field

The undistorted optic flow vector arising from a static three-dimensional point ***P***=(*X*,*Y*,*Z*) due to the movement of a monocular observer is described as

(Equation 2) $$v\left(P\right)=\frac{1}{Z}\left(\begin{array}{lll} -f & 0 & x\\ 0 & -f & y \end{array}\right)T+\left(\begin{array}{lll} \left(xy\right)/f & -\left(f+x^{2}/f\right) & y\\ f+y^{2}/f & -\left(xy\right)/f & -x \end{array}\right)\Omega =\left(\begin{array}{l} \dot{x}\\ \dot{y} \end{array}\right),$$

where $T={\left(T_{X},T_{Y},T_{Z}\right)}^{⊤}$ depicts the observer's translation and $\Omega ={\left({\text{Ω}}_{X},{\text{Ω}}_{Y},{\text{Ω}}_{Z}\right)}^{⊤}$ the rotation of the eye. This equation is the time derivative of the perspective projection

pr(P)=f(X/Z,Y/Z)=(x,y)

onto an image plane placed perpendicular to the line of sight in distance *f* and is used regularly to describe optic flow (Longuet-Higgins and Prazdny, 1980; Heeger and Jepson, 1992; Lappe and Rauschecker, 1995). Wearing PALs alters the path the light takes in the projection onto the image plane. To account for such effects we included distortions via the aforementioned functions F into the projection. Taking the derivative with respect to time of this new type of projection

$$pr_{d}\left(P\right)=\left(\begin{array}{l} F_{x}\left(pr\left(P\right)\right)\\ F_{y}\left(pr\left(P\right)\right) \end{array}\right)=\left(\begin{array}{l} F_{x}\left(x,y\right)\\ F_{y}\left(x,y\right) \end{array}\right)$$

results in the following equation for distorted optic flow,

(Equation 3) $$v_{d}\left(P\right)=\left(\begin{array}{l} {\dot{x}}_{d}\\ {\dot{y}}_{d} \end{array}\right)=\left(\begin{array}{ll} \frac{\partial F_{x}}{\partial x} & \frac{\partial F_{x}}{\partial y}\\ \frac{\partial F_{y}}{\partial x} & \frac{\partial F_{y}}{\partial y} \end{array}\right)\left(\begin{array}{l} \dot{x}\\ \dot{y} \end{array}\right).$$

Equations 2 and 3 were used to calculate the flow vector for a point P on the ground plane at image plane position pr(P) for the undistorted and *pr*_*d*_(***P***) for the distorted projection.

##### Heading estimation

The subspace algorithm (Heeger and Jepson, 1992) computes the likelihood of a set of candidate heading directions, the heading space, as a least-squares residual value for each of the candidate directions for a given flow field. This value describes how incongruous a particular direction is with the flow field. Finding the minimum in the residual surface of the heading space leads to the most suitable heading direction to explain the given flow field. In the model, the flow field is split into smaller, locally restricted patches that are evaluated separately. Finding the minimum of the sum of the emerging residual surfaces increases the robustness and uniqueness of the estimation.

A schematic illustration of the simulation process for a single flow field distorted due to the peripheral gaze condition can be seen in Figure 4. The flow field consists of four randomly placed patches, each containing 10 flow vectors (Figure 4A). The residual surfaces are calculated per patch and displayed after a logarithmic transformation for better discriminability (Figure 4B, left). Every single patch carries enough information for running the subspace algorithm and estimate the most likely heading for these flow vectors. Those headings are marked with red dots in the residual surfaces. The full model sums up all the residual surfaces and estimates the heading direction based on the summed residual landscape (Figure 4B, right).

The subspace algorithm finds the best-matching heading for a flow field that consists of observer translation and gaze rotation. It can be constrained to search only for combinations of gaze rotations that result from fixations of environmental objects or for gaze rotations that are free of torsion (Lappe and Rauschecker, 1995). In our simulations we included only the latter constraint.

For application of the subspace algorithm we needed to select points on the ground plane for which the flow vectors were calculated to constitute the flow field. For this, random locations for the centers of 50 patches in the visual field were selected and the size (area) of each patch set according to the following equation

(Equation 4) $$A={\left(1.04^{\circ }+0.61\varepsilon \right)}^{2}$$

with *ε* being the respective center point's eccentricity. This increase in patch size with eccentricity is based on similar findings in the visual system of primates and is well matched to the natural statistics of optic flow fields (Albright and Desimone, 1987; Calow and Lappe, 2007, 2008). Then, ten locations were chosen randomly inside each patch. At each location, the optic flow vector was calculated according to Equations (Equation 2), (Equation 3), assuming a point ***P*** on the ground plane that is projected onto this location, a translation T as pre-defined for the simulation, and a rotation **Ω** that nullifies the optic flow at the fixation point in the undistorted condition to simulate the tracking motion. Combining all of these vectors from within all patches results in the full flow field.

##### Simulation procedure

Aiming to simulate self-motion estimations based on optic flow we established the scene by placing a virtual monocular observer on a plane consisting of single points. Gaze was directed $20^{\circ }$ downwards onto the fixation point from a simulated eye height of 1.8 m. Translation was initialized $20^{\circ }$ to the left and to the right of that point and parallel to the floor with an added vertical heading angle between −3.5^∘^ and 3.5^∘^ in steps of 0.5^∘^. Rotation was calculated such that the gaze remains on the fixation point. To include the distortions we used the aforementioned functions F.

Candidate and heading directions were defined in retinal coordinates, relative to the gaze direction. Hence $\left(0^{\circ },20^{\circ }\right)$ describes the translation parallel to the floor toward the fixation point and was set to be the center of the set of candidate directions. That heading space covered an area of 70^∘^×20^∘^ in which all directions sampled in 0.25^∘^ steps horizontally and vertically were tested for their compatibility with the flow fields. This included all heading directions that were actually used in flow field calculations.

The maximum simulated FoV was set to be a circular area with radius $55^{\circ }$ in accordance to the specifications given by the VR-headset manufacturer. To explore differences in heading estimations based on different FoV sizes we further defined a set of effective FoV sizes to range from a FoV with radius 40^∘^ up to one with radius 55^∘^. These simulations used only patches of the flow field for which all the locations of the vectors in a patch are within the effective FoV.

Overall the complete simulation consisted of 50 separate runs, each containing the evaluation of flow fields that resulted from 90 combinations of heading directions and distortion conditions.

#### Experimental methods

To study human perception under the influence of PAL distortions in an isolated environment, we developed a simulation of optical distortions in VR. In a psychophysical experiment the self-motion scenario of moving along a virtual ground plane was recreated. PAL distortions were presented in two different conditions representing the two different directions of gaze through the progressive lens. The vertical heading perception of subjects was tested with a direction discrimination task.

##### Participants

Thirteen subjects (seven female and six male) aged between 21 and 28 years (mean = 24.0 years) participated in the study. None of the participants reported any ocular complication or any medical condition that would affect their normal vision or their motion judgements. There was no prior history of epilepsy or motion sickness.

##### Ethics

The study adhered to the tenets of the Helsinki Declaration (2013). The ethics authorisation to perform the measurements was granted by the Medical Faculty Human Research Ethics Committee from the University of Tuebingen. Prior to data collection, the experiment was explained in detail to the participants, and written informed consent was collected from each participant.

##### Distortion simulation in VR

The aforementioned ray-tracing function for an optical lens was be used in our VR framework to simulate the influence of optical distortions in a virtual environment. The distortion simulation was implemented in the game engine Unity (Unity Technologies, CA, USA) as a post processing shader (a program part manipulating the rendered image), which geometrically transforms the rendered image according to the mathematical transformation defined before. The pixel transformation is implemented as a fragment shader. For a pixel position in the output texture, the corresponding position in the input texture is needed. The inverse transformation function F^−1^, i.e., the mapping from distorted image to undistorted image, is used for this process. F^−1^ is approximated using a 2D polynomial fit of the ray traced distortion data: In Matlab, two functions were fitted, for the *x* and *y* component of F, so that F_*x*_^−1^(*x*_*d*_,*y*_*d*_) = *x* and F_*y*_^−1^(*x*_*d*_,*y*_*d*_) = *y*, where (*x*,*y*) are the defined object points for ray tracing and (*x*_*d*_,*y*_*d*_) corresponding distorted points, as perceived through a progressive lens.

Before applying the function, the Unity texture coordinates (*u*,*v*)∈[0,1]×[0,1] had to be scaled and shifted to points (*x*,*y*) in the corresponding image plane considering the virtual cameras horizontal and vertical FoV. The center of the rendered left and right eye image textures are not exactly the center of visual field, corresponding to the center of the ray tracing simulations. This vertical and horizontal displacement is accessible from the projection matrices of the left and right eye virtual cameras in Unity. The coordinates (*x*,*y*) were shifted by this displacement $\left({\text{Δ}}_{x},{\text{Δ}}_{y}\right)$ so that the origin (0,0) corresponds to the center of the simulated distortion mesh

(Equation 5) $$\left(\begin{array}{l} x\\ y \end{array}\right)=\left(\begin{array}{l} 2\left(u-0.5\right)\text{tan}\left(\frac{{\text{FoV}}_{\text{hor}}}{2}\right)\\ 2\left(v-0.5\right)\text{tan}\left(\frac{{\text{FoV}}_{\text{ver}}}{2}\right) \end{array}\right)-\left(\begin{array}{l} {\text{Δ}}_{x}\\ {\text{Δ}}_{y} \end{array}\right)$$

To determine the corresponding pixel position in the undistorted image (*x*,*y*), the inverse transformation is applied to (*x*_*d*_,*y*_*d*_):

(Equation 6) $$\left(x,y\right)=F^{-1}\left(x_{d},y_{d}\right)$$

The image plane coordinates (*x*,*y*) are now mapped back to texture coordinates (*u*,*v*) of the original undistorted texture:

(Equation 7) $$u=\frac{x+\text{Δ}x}{2\text{tan}\left(\frac{{\text{FoV}}_{\text{hor}}}{2}\right)}+0.5$$

(Equation 8) $$v=\frac{y+\text{Δ}y}{2\text{tan}\left(\frac{{\text{FoV}}_{\text{ver}}}{2}\right)}+0.5$$

Unity then automatically interpolated the value of the pixel (*u*_*d*_,*v*_*d*_) in the output texture from pixels (*u*,*v*) in the input texture. This results in the distorted image.

The shader-based coordinate scaling and pixel transformation was validated by comparing the rendered output with a distorted texture precomputed in using the same transformation function.

##### Stimulus creation and VR setup

The virtual environment for the experiment was implemented using Unity and presented to participants using an HTC Vive Pro Eye VR headset (HTC Corporation, Taoyuan, Taiwan). The manufacturer claims a FoV of the VR headset of 110^∘^, although the effectively visible FoV is usually smaller, depending on the individual subject's eye position and placement of the headset on the head. Optic flow was induced by a moving virtual ground plane with a perlin noise texture. The background above the horizon was uniformly gray. To present the same optic flow to each subject, the virtual ground plane was fixed relative to the subject's view with the virtual eye height of 1.8 m. The direction of the virtual camera was 20^°^ downwards and 20^°^ left or right (randomized between subjects) relative to the direction of motion. The subject's virtual position relative to the floor and motion direction are illustrated in Figure 2. Position and orientation tracking of the VR headset was turned off for this experiment, in order for the presented stimulus not to be influenced by a change of position or orientation of the subject. Apart from the ground plane, only a dot was shown in the center of the visual field as a fixation target.

##### Procedure

Subjects were introduced to the experiment procedure before putting on the VR headset. They performed the experiment in a seated position with their head placed on a chin rest to maintain a stable head position. At the beginning of a trial, only a centered dot as fixation target was visible, while the ground plane was not shown yet. Subjects were instructed to fixate the fixation target. Compliance was controlled by the eye tracker of the Vive Pro Eye headset. Subjects pressed a key on a keyboard to start the trial. The motion phase of the trial started only if gaze was within 2^∘^ to the fixation target. After the keypress, the ground plane faded in within 0.2s while the fixation point faded out. The fade out of the fixation point ensured that subjects could perform involuntary tracking motion during the later presented flow (Miles and Busettini, 1992; Lappe et al., 1998; Angelaki and Hess, 2005). Then the actual motion started. It simulated a 0.3s translation over the ground plane at a speed of $2{\text{ms}}^{-1}$ with a vertical component (heading angle) between −3.5^°^ and 3.5^°^ in steps of 0.5^°^.

Afterward, the ground plane disappeared and subjects reported their perceived vertical heading direction by pressing the ’up’ key on the keyboard when they felt lifting up and the ’down’ key when they felt sinking. The next trial started after the subjects gave their response. In one session, all three distortion conditions - undistorted, central view, and peripheral view - were presented. All motion directions were performed 12 times for all distortion conditions leading to 540 trials in total. The order of presented distortions, as well as heading angles, was randomized.

### Quantification and statistical analysis

To measure the influence of the distortions on heading perception, the vertical translation angle perceived as a motion parallel to the floor was determined for each of the three distortion conditions. For each subject and each distortion condition, first, the percentage of answers upwards was computed depending on the vertical angle of the simulated motion relative to the ground plane.

These datasets were then fitted with a cumulative normal distribution function with free but equal asymptotes using Psignifit (Schütt et al., 2016). The 50% point of the fit function was used as the point of subjective equality (PSE). It corresponds to the vertical motion angle that is perceived as a movement parallel to the floor by the subject in the specific condition.

To identify the influence of the simulated distortions, we compared the PSEs in the two distorted conditions to the undistorted condition by calculating the difference ΔPSE. As statistical analysis, one-sample *t*-tests were used with ΔPSE to test the presence of a significant shift of perceived heading.

## Data Availability

Experimental data and simulation results reported in this paper will be shared by the lead contact upon request. The model simulation code is accessible on github.com. Any additional information required to reanalyze the data reported in this paper is available from the lead contact upon request.
